# Urinary phytoestrogens and hyperuricemia risk in a nationally representative population: an effect modified by smoking status

**DOI:** 10.1038/s41598-026-45911-5

**Published:** 2026-05-06

**Authors:** Chunliang Liu, Zhihua Yang, Huizhi Feng, Mengting Liu, Mengrou Zhai, Jiaying Yuan, Jinliang Niu

**Affiliations:** 1 Department of Gastroenterology, The Second Hospital of Shanxi Medical University, 382 Wuyi Road, Taiyuan, Shanxi China; 2 The Second Clinical Medical College, Shanxi Medical University, Taiyuan, Shanxi China; 3 Department of Gastroenterology, Shanxi Province Cancer Hospital, Taiyuan, Shanxi China; 4 Department of Radiology, The Second Hospital of Shanxi Medical University, 382 Wuyi Road, Taiyuan, Shanxi China

**Keywords:** Phytoestrogens, Hyperuricemia, Cigarette smoking, Epidemiology, Biomarkers, Diseases, Environmental sciences, Medical research, Risk factors

## Abstract

Evidence on the relationship between phytoestrogen exposure and hyperuricemia remains limited and inconsistent. We investigated the associations of urinary phytoestrogens with serum uric acid concentrations and hyperuricemia risk in a nationally representative U.S. population and explored the potential effect modification by smoking status. This study included 8,160 participants aged 20 years and older from the 1999–2010 National Health and Nutrition Examination Survey (NHANES). Urinary concentrations of phytoestrogens were determined using high-performance liquid chromatography–tandem mass spectrometry and adjusted for urinary creatinine. Multivariable linear and logistic regression models were employed to evaluate associations with serum uric acid levels and hyperuricemia risk, while restricted cubic splines were applied to examine nonlinear dose-response relationships. Following multivariable adjustment, higher urinary concentrations of total phytoestrogens, isoflavones, lignans, and several specific compounds were linked to a lower risk of hyperuricemia. The strongest associations were exhibited for microbial metabolites, including equol [OR (95% CI) for quartile 4 vs. 1: 0.46 (0.35, 0.59)], enterolactone [0.60 (0.49, 0.74)], and O-desmethylangolensin (O-DMA) [0.64 (0.51, 0.79)]. These inverse associations were more pronounced among current smokers (p-interaction for total phytoestrogens and isoflavones = 0.019) and largely followed L-shaped dose–response patterns. This study suggested that elevated urinary phytoestrogen levels were associated with a reduced risk of hyperuricemia in U.S. adults, particularly among current smokers. If confirmed by prospective studies, phytoestrogen-rich diets may represent a promising strategy for hyperuricemia prevention.

**Supplementary Information:**

The online version contains supplementary material available at 10.1038/s41598-026-45911-5.

## Introduction

Hyperuricemia is a common metabolic disorder defined by elevated serum uric acid levels, typically above 360 µmol/L in women and 420 µmol/L in men^1^. Its prevalence has been rising worldwide, especially in developed and rapidly urbanizing regions, making it an increasing public health concern^1^. Beyond its role as the primary cause of gout, hyperuricemia is considered an independent risk factor for several chronic conditions, including hypertension, cardiovascular disease, metabolic syndrome, and chronic kidney disease^2^. Established risk factors for hyperuricemia include obesity, high-purine diet, and alcohol consumption^3,4^; however, the growing disease burden highlights the need to identify more modifiable lifestyle and dietary factors, particularly dietary components in epidemiological studies.

Phytoestrogens are a class of plant-derived polyphenolic compounds with structural similarity to human estrogens and are mainly classified as isoflavones and lignans^5^. Isoflavones, abundant in soy products, include daidzein and genistein. Notably, daidzein can be further metabolized by gut microbiota into O-desmethylangolensin (O-DMA) and equol^6,7^. Lignans, present in seeds, whole grains, and berries, are converted by intestinal bacteria into enterodiol and enterolactone^8^. These microbial transformations are critical in determining the bioavailability and biological activity of phytoestrogens. Accordingly, urinary concentrations of these phytoestrogens serve as an integrated biomarker of internal exposure, capturing both dietary intake and host microbial metabolic activity, and thereby providing a more comprehensive and objective assessment than dietary questionnaires^9^.

Phytoestrogens, known for their potential benefits in cardiometabolic health, have also been hypothesized to influence uric acid metabolism. Clinical observations have indicated that exogenous estrogen administration can lower plasma uric acid levels^10^, and cross-sectional analyses have identified an inverse relationship between serum estradiol and hyperuricemia risk^11^. These findings suggest phytoestrogens, as estrogen-like compounds, may modulate uric acid homeostasis through estrogen receptor–mediated pathways. Supporting evidence from animal studies demonstrates that supplementation with soy isoflavones such as daidzein and genistein effectively reduced serum uric acid concentrations^12^. At the molecular level, in vitro studies revealed that these compounds inhibit xanthine oxidase, the key enzyme in purine catabolism, as well as cyclooxygenase-2, thereby attenuating both uric acid production and inflammatory responses^13–15^.

Epidemiological evidence on the associations between phytoestrogen exposure and hyperuricemia remains inconsistent. Cross-sectional studies in Chinese and U.S. populations have reported inverse associations between dietary intake of soy isoflavones (including daidzein and genistein) and hyperuricemia risk^16,17^. Another study found that equol producers exhibited lower serum uric acid levels, whereas O-DMA producers did not^18^. In contrast, a recent meta-analysis of long-term clinical studies reported null associations between soy isoflavones and uric acid concentrations^12^. These discrepancies may reflect differences in study design, dietary assessment accuracy, interindividual variability in microbial metabolism of phytoestrogens, and limited sample sizes. Therefore, studies using objective exposure biomarkers in large, nationally representative populations are warranted to clarify these associations.

The present study aimed to evaluate the associations of urinary total phytoestrogens, four isoflavones (i.e., genistein, daidzein, O-DMA, and equol), and two lignans (i.e., enterodiol and enterolactone) with serum uric acid levels and hyperuricemia risk. In addition, given that cigarette smoking is a known modulator of estrogenic activity and uric acid handling^19,20^, we further assessed whether smoking status modified the observed associations.

## Methods

### Study population

This study utilized data from the National Health and Nutrition Examination Survey (NHANES) conducted between 1999 and 2010, which were the survey cycles that included measurements of both urinary phytoestrogens and serum uric acid. All analytical measurements were obtained from the NHANES study and were not collected by the authors themselves. NHANES is an ongoing national program of cross-sectional surveys assessing the health and nutritional characteristics of the U.S. civilian, noninstitutionalized population through a complex, multistage probability sampling method. Details of the survey design and operational procedures have been published elsewhere^21^.

During the 1999–2010 cycles, 62,160 individuals were recruited. We sequentially excluded individuals aged < 20 years (*n* = 29,696), those missing weight data (*n* = 22,320), individuals without information on covariates (*n* = 959), those lacking urinary phytoestrogens (*n* = 643) or serum uric acid measurements (*n* = 372), and those with hypouricemia (*n* = 10). After these exclusions, 8,160 adults were retained for the final analysis (Supplementary Fig. 1). All NHANES study protocols were approved by the National Center for Health Statistics Research Ethics Review Board, and all participants provided written informed consent^22^.

### Serum uric acid measurement and hyperuricemia classification

Serum uric acid concentrations were measured by a colorimetric method using the Beckman Synchron LX20 analyzer in 2007 and earlier, and the Beckman Coulter UniCel^®^ DxC800 in subsequent survey cycles. Hyperuricemia was determined according to serum uric acid thresholds of ≥ 420 µmol/L for men and ≥ 360 µmol/L for women^1^. Hypouricemia was defined as a serum uric acid concentration of < 119 µmol/L^23^.

### Urinary phytoestrogen and creatinine measurement

Urine samples were processed following standardized NHANES laboratory protocols. Phytoestrogen concentrations were quantified by high-performance liquid chromatography coupled with atmospheric pressure photoionization tandem mass spectrometry (HPLC-APPI-MS/MS), a method validated for high sensitivity and specificity. Urinary creatinine was quantified using the Jaffe reaction (Beckman CX3 analyzer) through 2006 and an enzymatic method (Roche ModP analyzer) thereafter. To ensure comparability across this methodological change, creatinine values from the 2005–2006 cycle were adjusted to align with subsequent cycles using a piecewise square-root transformation^24^. Finally, phytoestrogen concentrations were adjusted for urinary creatinine (µg/g creatinine) to minimize the impact of urine dilution^25^. Total isoflavones were calculated as the sum of genistein, daidzein, O-DMA, and equol. Total lignans were calculated as the sum of enterodiol and enterolactone. Total phytoestrogens represented the combined concentrations of all compounds.

### Data collection

Information on covariates was obtained using standardized questionnaires and physical examinations. Demographic characteristics comprised age, sex, and race/ethnicity (categorized as non-Hispanic White, non-Hispanic Black, Mexican American, and other). Lifestyle-related indicators included body mass index (< 25, 25–30, and ≥ 30 kg/m^2^), educational level (high school graduate or below vs. some college or above), cigarette smoking (never, former, or current), and alcohol consumption (never, former, or current). Medical history variables included self-reported diagnoses of hypertension, diabetes, cancer, cardiovascular disease, chronic kidney disease, and liver disease.

### Statistical analysis

All statistical analyses were performed with SAS survey procedures in accordance with the complex, multistage sampling design of NHANES to achieve national representativeness. Participant characteristics were summarized across quartiles of urinary total phytoestrogen concentrations. Continuous variables were reported as weighted means with standard errors and evaluated through sampling-weighted analysis of variance (ANOVA), while qualitative variables were expressed as unweighted counts and weighted proportions and examined using the Rao–Scott chi-square test.

To examine the associations of urinary phytoestrogen concentrations with serum uric acid levels, multiple linear regression models were applied, with log-transformed serum uric acid as the dependent variable and log-transformed urinary phytoestrogen concentrations as the independent variables. Three models were constructed: Model 1 had no covariate adjustment; Model 2 controlled for demographic and behavioral variables (age, sex, race/ethnicity, body mass index, education level, cigarette smoking, and alcohol intake); and Model 3 additionally accounted for chronic conditions including hypertension, diabetes, cancer, cardiovascular disease, chronic kidney disease, and liver disease. Regression coefficients (β) and 95% confidence intervals (CIs) were calculated.

Associations between urinary phytoestrogen levels and hyperuricemia were evaluated using multivariable logistic regression. Odds ratios (ORs) and 95% CIs were calculated for each quartile of urinary phytoestrogens, using the lowest quartile as reference. Linear trends across quartiles were assessed by treating the median value of each quartile as a continuous variable. Hyperuricemia risk refers to the likelihood of meeting established sex-specific diagnostic thresholds. Stratified analyses were undertaken according to cigarette smoking status (never/former vs. current smokers), age (< 50 years vs. ≥50 years), sex (male vs. female), body mass index (< 30 kg/m^2^ vs. ≥30 kg/m^2^), race/ethnicity (non-Hispanic White vs. other races), and history of cancer (no vs. yes), and multiplicative interaction terms were incorporated into the models to examine potential statistical interactions.

Restricted cubic spline models with three knots at the 5th, 50th, and 95th percentiles of urinary phytoestrogen concentrations were applied to evaluate potential nonlinear dose–response relationships with hyperuricemia risk. The median concentration was used as the reference point in these models. Sensitivity analyses were conducted after excluding participants with abnormally high serum creatinine concentrations (≥ 97.5th percentile), and the results remained largely consistent across models. All statistical tests were two-tailed, and a p-value below 0.05 was regarded as statistically significant.

## Results

### Characteristics of participants

A descriptive overview of the entire study population is presented in Supplementary Table 1. Table 1 presents participant characteristics stratified by quartiles of urinary phytoestrogen concentrations. Individuals with higher urinary total phytoestrogen levels were more likely to be older, female, non-Hispanic White, leaner (BMI < 25 kg/m^2^), more highly educated, and more often never-smokers (all *p* < 0.001). In addition, cancer and cardiovascular disease were more prevalent among participants with higher urinary phytoestrogen levels (both *p* < 0.05). Notably, both the prevalence of hyperuricemia and serum uric acid concentrations declined progressively across increasing quartiles of urinary total phytoestrogens (both *p* < 0.001).

**Table 1:** Characteristics of participants by quartiles of urinary total phytoestrogens.

Characteristics	Urinary total phytoestrogens (µg/g creatinine)				
	Q1 (< 317.2) *n* = 2,082	Q2 (317.2–700.0) *n* = 2,078	Q3 (700.0-1459.5) *n* = 2,055	Q4 (≥ 1459.5) *n* = 1,945	*p*-value
Age, mean (SE)	42.6 (0.37)	45.7 (0.47)	47.9 (0.47)	50.2 (0.59)	**< 0.001**
Sex [n (weighted %)]					**< 0.001**
Male	1,164 (58.31%)	1,058 (51.28%)	973 (46.66%)	782 (38.70%)	
Female	918 (41.69%)	1,020 (48.72%)	1,082 (53.34%)	1,163 (61.30%)	
Race/Ethnicity [n (weighted %)]					**< 0.001**
White, Non-Hispanic	903 (65.93%)	991 (70.49%)	1,072 (73.47%)	1,145 (78.47%)	
Black, Non-Hispanic	488 (13.78%)	419 (11.49%)	382 (10.19%)	239 (6.00%)	
Mexican American	439 (8.38%)	477 (8.93%)	418 (8.02%)	357 (6.24%)	
Other Race	252 (11.90%)	191 (9.10%)	183 (8.31%)	204 (9.29%)	
BMI (kg/m^2^) [n (weighted %)]					**< 0.001**
< 25	490 (25.57%)	546 (28.52%)	641 (34.55%)	741 (42.13%)	
25–30	704 (32.01%)	739 (34.51%)	727 (32.39%)	668 (32.68%)	
≥ 30	888 (42.42%)	793 (36.97%)	687 (33.06%)	536 (25.19%)	
Education level [n (weighted %)]					**< 0.001**
High school graduate or below	1,216 (50.72%)	1,168 (47.09%)	1,053 (42.55%)	855 (33.39%)	
Some college or above	866 (49.28%)	910 (52.91%)	1,002 (57.45%)	1,090 (66.61%)	
Cigarette smoking [n (weighted %)]					**< 0.001**
Never smoker	1,008 (47.18%)	1,054 (49.62%)	1,099 (52.77%)	1,095 (55.29%)	
Former smoker	464 (21.21%)	532 (23.31%)	564 (26.62%)	564 (29.08%)	
Current smoker	610 (31.61%)	492 (27.07%)	392 (20.60%)	286 (15.63%)	
Alcohol intake [n (weighted %)]					0.431
Never drinker	261 (9.39%)	280 (10.52%)	291 (12.05%)	282 (10.64%)	
Former drinker	312 (15.13%)	341 (14.65%)	324 (14.20%)	297 (13.66%)	
Current drinker	1,509 (75.48%)	1,457 (74.83%)	1,440 (73.74%)	1,366 (75.69%)	
Hypertension [n (weighted %)]	636 (28.32%)	664 (29.02%)	690 (29.95%)	671 (28.77%)	0.787
Diabetes [n (weighted %)]	196 (7.22%)	208 (7.16%)	226 (8.04%)	218 (7.55%)	0.756
Cancer [n (weighted %)]	126 (5.86%)	181 (8.65%)	192 (8.72%)	240 (11.68%)	**< 0.001**
Cardiovascular disease [n (weighted %)]	171 (6.70%)	207 (8.68%)	239 (9.63%)	224 (8.22%)	**0.036**
Chronic kidney disease [n (weighted %)]	57 (2.25%)	36 (1.46%)	45 (1.77%)	46 (1.43%)	0.296
Liver condition [n (weighted %)]	70 (3.35%)	68 (3.57%)	52 (2.49%)	59 (3.03%)	0.399
Hyperuricemia [n (weighted %)]	456 (22.15%)	383 (17.98%)	354 (15.99%)	289 (13.18%)	**< 0.001**
Serum uric acid (µmol/L) [mean (SE)]	329.66 (2.27)	314.44 (1.92)	306.82 (2.29)	293.66 (2.23)	**< 0.001**

Bold p-values indicate statistical significance.

### Associations of urinary phytoestrogens with serum uric acid levels

The results of multiple linear regression analyses are shown in Table 2. After adjustment for potential confounders (model 2 and 3), urinary levels of total and most individual phytoestrogens, except enterodiol, were inversely and linearly associated with serum uric acid levels (all *p* ≤ 0.002). In the fully adjusted model (Model 3), the strongest inverse associations were observed for equol (β: -0.028; 95% CI: -0.036, -0.021), enterolactone (β: -0.027; 95% CI: -0.034, -0.020), O-DMA (β: -0.024; 95% CI: -0.032, -0.017), and total phytoestrogens (β: -0.023; 95% CI: -0.030, -0.017).

**Table 2:** Multiple linear regression analysis of the associations between urinary phytoestrogens and serum uric acid level^*^.

Urinary total phytoestrogens	Serum uric acid (µmol/L)		
(µg/g creatinine)	Model 1	Model 2	Model 3
Total phytoestrogens			
β (95% CI)	-0.054 (-0.061, -0.046)	-0.024 (-0.030, -0.018)	-0.023 (-0.030, -0.017)
p	**< 0.001**	**< 0.001**	**< 0.001**
Total isoflavones			
β (95% CI)	-0.040 (-0.048, -0.031)	-0.020 (-0.028, -0.013)	-0.020 (-0.027, -0.013)
p	**< 0.001**	**< 0.001**	**< 0.001**
Total lignans			
β (95% CI)	-0.047 (-0.055, -0.039)	-0.022 (-0.028, -0.015)	-0.021 (-0.027, -0.014)
p	**< 0.001**	**< 0.001**	**< 0.001**
Genistein			
β (95% CI)	-0.027 (-0.036, -0.019)	-0.013 (-0.021, -0.005)	-0.013 (-0.021, -0.005)
p	**< 0.001**	**0.001**	**0.002**
Daidzein			
β (95% CI)	-0.035 (-0.044, -0.026)	-0.020 (-0.027, -0.013)	-0.020 (-0.028, -0.013)
p	**< 0.001**	**< 0.001**	**< 0.001**
O-DMA			
β (95% CI)	-0.045 (-0.054, -0.035)	-0.025 (-0.032, -0.017)	-0.024 (-0.032, -0.017)
p	**< 0.001**	**< 0.001**	**< 0.001**
Equol			
β (95% CI)	-0.046 (-0.054, -0.038)	-0.029 (-0.036, -0.022)	-0.028 (-0.036, -0.021)
p	**< 0.001**	**< 0.001**	**< 0.001**
Enterodiol			
β (95% CI)	-0.027 (-0.035, -0.019)	-0.003 (-0.010, 0.003)	-0.003 (-0.010, 0.003)
p	**< 0.001**	0.349	0.336
Enterolactone			
β (95% CI)	-0.049 (-0.058, -0.041)	-0.028 (-0.035, -0.021)	-0.027 (-0.034, -0.020)
p	**< 0.001**	**< 0.001**	**< 0.001**

CI: confidence interval; O-DMA: O-desmethylangolensin.

* β represents the partial regression coefficient, showing the change in log-transformed serum uric acid (µmol/L) per unit increase in log-transformed urinary phytoestrogens (µg/g creatinine).

Model 1: Unadjusted for covariates. Model 2: Adjusted for age, sex, race, body mass index, education level, cigarette smoking, and alcohol intake. Model 3: Based on Model 2, with further adjustment for hypertension, diabetes, cancer, cardiovascular disease, chronic kidney disease, and liver condition.

Bold p-values indicate statistical significance.

### Associations of urinary phytoestrogens with hyperuricemia

Multivariable logistic regression analyses are shown in Table 3. After full adjustment (model 3), the risk of hyperuricemia declined progressively across quartiles of urinary total phytoestrogens, isoflavones, lignans, and most specific compounds (all p-trend < 0.05), whereas no significant trends were observed for genistein and enterodiol. For example, ORs (95% CIs) for the second, third, and fourth quartiles of urinary equol relative to the lowest quartile were 0.65 (0.52, 0.83), 0.58 (0.46, 0.73), and 0.46 (0.35, 0.59), respectively (p-trend < 0.001). The corresponding values for urinary genistein were 0.72 (0.58, 0.90), 0.79 (0.66, 0.96), and 0.72 (0.59, 0.88), with no significant trend observed (p-trend = 0.065).

**Table 3:** ORs (95% CIs) for hyperuricemia in relation to urinary phytoestrogens^*^.

Urinary phytoestrogens	Quartile of urinary phytoestrogens (µg/g creatinine)				
	1	2	3	4	*p*-trend
Total phytoestrogens					
n^#^	456/1,626	383/1,695	354/1,701	289/1,656	
Concentrations (range)	< 317.2	317.2–700.0	700.0-1459.5	≥ 1459.5	
Model 1	Reference	0.77 (0.65, 0.92)	0.67 (0.55, 0.82)	0.53 (0.44, 0.65)	**< 0.001**
Model 2	Reference	0.76 (0.63, 0.93)	0.67 (0.54, 0.84)	0.59 (0.48, 0.73)	**< 0.001**
Model 3	Reference	0.77 (0.63, 0.93)	0.67 (0.53, 0.84)	0.60 (0.49, 0.75)	**< 0.001**
Total isoflavones					
n^#^	458/1,729	351/1,642	344/1,650	329/1,657	
Concentrations (range)	< 43.5	43.5-102.6	102.6-316.3	≥ 316.3	
Model 1	Reference	0.72 (0.62, 0.84)	0.71 (0.58, 0.86)	0.59 (0.49, 0.70)	**< 0.001**
Model 2	Reference	0.68 (0.57, 0.81)	0.66 (0.54, 0.81)	0.59 (0.49, 0.71)	**< 0.001**
Model 3	Reference	0.67 (0.56, 0.80)	0.65 (0.52, 0.81)	0.58 (0.48, 0.70)	**< 0.001**
Total lignans					
n^#^	467/1,578	368/1,677	343/1,748	304/1,675	
Concentrations (range)	< 158.3	158.3-433.8	433.8-980.7	≥ 980.7	
Model 1	Reference	0.70 (0.59, 0.83)	0.61 (0.51, 0.74)	0.57 (0.47, 0.70)	**< 0.001**
Model 2	Reference	0.72 (0.60, 0.86)	0.62 (0.50, 0.75)	0.64 (0.52, 0.78)	**0.001**
Model 3	Reference	0.72 (0.60, 0.86)	0.62 (0.51, 0.76)	0.64 (0.52, 0.80)	**0.002**
Genistein					
n^#^	402/1655	367/1672	363/1675	350/1676	
Concentrations (range)	< 8.8	8.8–23.6	23.6–78.6	≥ 78.6	
Model 1	Reference	0.76 (0.62, 0.93)	0.86 (0.72, 1.03)	0.72 (0.60, 0.86)	**0.012**
Model 2	Reference	0.74 (0.60, 0.92)	0.81 (0.68, 0.98)	0.73 (0.60, 0.89)	0.050
Model 3	Reference	0.72 (0.58, 0.90)	0.79 (0.66, 0.96)	0.72 (0.59, 0.88)	0.065
Daidzein					
n^#^	424/1,711	370/1,622	341/1,680	347/1,665	
Concentrations (range)	< 17.5	17.5–49.9	49.9-170.1	≥ 170.1	
Model 1	Reference	0.84 (0.70, 1.02)	0.72 (0.59, 0.88)	0.70 (0.59, 0.83)	**0.002**
Model 2	Reference	0.82 (0.66, 1.03)	0.68 (0.55, 0.84)	0.70 (0.58, 0.84)	**0.006**
Model 3	Reference	0.81 (0.65, 1.01)	0.66 (0.53, 0.82)	0.68 (0.56, 0.82)	**0.005**
O-DMA					
n^#^	462/1,771	365/1,697	326/1,603	329/1,607	
Concentrations (range)	< 0.7	0.7–3.4	3.4–18.9	≥ 18.9	
Model 1	Reference	0.77 (0.62, 0.96)	0.71 (0.57, 0.88)	0.64 (0.53, 0.78)	**0.001**
Model 2	Reference	0.71 (0.57, 0.88)	0.66 (0.52, 0.83)	0.64 (0.52, 0.79)	**0.013**
Model 3	Reference	0.71 (0.57, 0.87)	0.65 (0.51, 0.83)	0.64 (0.51, 0.79)	**0.012**
Equol					
n^#^	537/1,752	370/1,716	326/1,629	249/1,581	
Concentrations (range)	< 3.3	3.3–7.1	7.1–15.0	≥ 15.0	
Model 1	Reference	0.71 (0.57, 0.87)	0.62 (0.51, 0.77)	0.48 (0.38, 0.60)	**< 0.001**
Model 2	Reference	0.66 (0.52, 0.83)	0.58 (0.46, 0.72)	0.46 (0.35, 0.59)	**< 0.001**
Model 3	Reference	0.65 (0.52, 0.83)	0.58 (0.46, 0.73)	0.46 (0.35, 0.59)	**< 0.001**
Enterodiol					
n^#^	434/1,758	359/1,713	375/1,618	314/1,589	
Concentrations (range)	< 16.1	16.1–41.9	41.9-101.4	≥ 101.4	
Model 1	Reference	0.85 (0.70, 1.03)	0.84 (0.68, 1.02)	0.80 (0.63, 1.00)	0.116
Model 2	Reference	0.84 (0.68, 1.03)	0.84 (0.68, 1.03)	0.91 (0.73, 1.14)	0.874
Model 3	Reference	0.83 (0.68, 1.02)	0.83 (0.67, 1.02)	0.90 (0.72, 1.13)	0.799
Enterolactone					
n^#^	469/1,518	370/1,711	343/1,755	300/1,694	
Concentrations (range)	< 98.6	98.6-351.2	351.2-864.4	≥ 864.4	
Model 1	Reference	0.67 (0.56, 0.80)	0.59 (0.49, 0.71)	0.54 (0.44, 0.65)	**< 0.001**
Model 2	Reference	0.67 (0.55, 0.80)	0.59 (0.49, 0.73)	0.59 (0.48, 0.72)	**< 0.001**
Model 3	Reference	0.67 (0.56, 0.81)	0.60 (0.49, 0.74)	0.60 (0.49, 0.74)	**< 0.001**

OR: odds ratio; CI: confidence interval; O-DMA: O-desmethylangolensin.

* Model 1: Unadjusted for covariates. Model 2: Adjusted for age, sex, race, body mass index, education level, cigarette smoking, and alcohol intake. Model 3: Based on Model 2, with further adjustment for hypertension, diabetes, cancer, cardiovascular disease, chronic kidney disease, and liver condition.

^#^ n: the number of individuals with hyperuricemia/normouricemia.

Bold p-values indicate statistical significance.

### Associations between urinary phytoestrogens and hyperuricemia stratified by cigarette smoking and other factors

As shown in Table 4, higher urinary levels of phytoestrogens (except genistein and enterodiol) were significantly and inversely associated with hyperuricemia among both never/former smokers and current smokers, with consistently stronger associations observed among current smokers in fully adjusted models. Among never/former smokers, participants in the highest quartile of total and specific phytoestrogens had approximately 28–53% lower odds of hyperuricemia than those in the lowest quartile (ORs = 0.47–0.72, all *p* < 0.05). In contrast, these inverse associations were markedly stronger among current smokers: participants in the highest quartile of total phytoestrogens, total isoflavones, and equol exhibited 60–67% lower odds of hyperuricemia compared with the lowest quartile (ORs = 0.33–0.40, all p-trend < 0.01). Genistein was inversely related to hyperuricemia only among current smokers (p-trend < 0.05) but not among never/former smokers (p-trend > 0.05), whereas enterodiol showed no significant association in either subgroup. Significant interactions were identified for total phytoestrogens and total isoflavones (both *p* = 0.019), indicating a clear effect modification by smoking status.

In addition to smoking status, stratified analyses by other factors, including age, sex, body mass index, race/ethnicity, and history of cancer, are presented in Supplementary Tables 2–6. A significant interaction between enterodiol and sex was observed (p-interaction < 0.001). Specifically, higher urinary enterodiol levels were inversely associated with hyperuricemia among males [OR (95% CI) for quartile 4 vs. 1: 0.66 (0.50–0.88), p-trend = 0.025], but positively associated among females [OR (95% CI): 1.40 (1.00–1.98), p-trend = 0.036] (Supplementary Table 3). In contrast, stratified analyses by age, body mass index, race/ethnicity, and cancer history showed results generally consistent with the overall findings, with no evidence of significant effect modification (Supplementary Tables 2, 4–6).

**Table 4:** Hyperuricemia risk in relation to urinary phytoestrogens stratified by cigarette smoking.

Urinary phytoestrogens(µg/g creatinine)	Never or former smoking		Current smoking		p-interaction
	n^*^	OR^#^ (95% CI)	n^*^	OR^#^ (95% CI)	
Total phytoestrogens					**0.019**
Q1: <317.2	333/1,139	Reference	123/487	Reference	
Q2: 317.2-700.0	313/1,273	0.81 (0.65, 1.01)	70/422	0.65 (0.47, 0.89)	
Q3: 700.0-1459.5	293/1,370	0.69 (0.53, 0.89)	61/331	0.64 (0.42, 0.98)	
Q4: ≥1459.5	261/1,398	0.67 (0.53, 0.85)	28/258	0.33 (0.19, 0.57)	
p-trend		**0.006**		**<0.001**	
Total isoflavones					**0.019**
Q1: <43.5	355/1,297	Reference	103/432	Reference	
Q2: 43.5-102.6	283/1,272	0.68 (0.55, 0.83)	68/370	0.64 (0.43, 0.95)	
Q3: 102.6-316.3	280/1,311	0.63 (0.51, 0.79)	64/339	0.71 (0.44, 1.14)	
Q4: ≥316.3	282/1,300	0.64 (0.51, 0.80)	47/357	0.37 (0.23, 0.60)	
p-trend		**0.029**		**< 0.001**	
Total lignans					0.131
Q1: <158.3	345/1,062	Reference	122/516	Reference	
Q2: 158.3-433.8	289/1,281	0.68 (0.54, 0.84)	79/396	0.84 (0.58, 1.22)	
Q3: 433.8-980.7	292/1,395	0.63 (0.50, 0.79)	51/353	0.58 (0.37, 0.89)	
Q4: ≥980.7	274/1,442	0.66 (0.53, 0.81)	30/233	0.51 (0.29, 0.91)	
p-trend		**0.013**		**0.012**	
Genistein					0.080
Q1: <8.8	310/1,265	Reference	92/390	Reference	
Q2: 8.8-23.6	300/1,314	0.74 (0.56, 0.97)	67/358	0.70 (0.42, 1.18)	
Q3: 23.6-78.6	297/1,309	0.84 (0.68, 1.03)	66/366	0.63 (0.38, 1.03)	
Q4: ≥78.6	293/1,292	0.81 (0.64, 1.02)	57/384	0.50 (0.30, 0.83)	
p-trend		0.490		**0.030**	
Daidzein					0.150
Q1: <17.5	321/1,280	Reference	103/431	Reference	
Q2: 17.5-49.9	303/1,259	0.82 (0.63, 1.07)	67/363	0.79 (0.52, 1.19)	
Q3: 49.9-170.1	284/1,335	0.67 (0.53, 0.83)	57/345	0.62 (0.39, 0.97)	
Q4: ≥170.1	292/1,306	0.72 (0.58, 0.91)	55/359	0.53 (0.34, 0.82)	
p-trend		0.057		**0.015**	
O-DMA					0.413
Q1: <0.7	347/1,285	Reference	115/486	Reference	
Q2: 0.7-3.4	299/1,301	0.73 (0.57, 0.93)	66/396	0.66 (0.43, 1.00)	
Q3: 3.4-18.9	269/1,264	0.65 (0.49, 0.86)	57/339	0.65 (0.43, 0.99)	
Q4: ≥18.9	285/1,330	0.65 (0.52, 0.83)	44/277	0.54 (0.35, 0.84)	
p-trend		**0.036**		0.055	
Equol					0.430
Q1: <3.3	409/1,282	Reference	128/470	Reference	
Q2: 3.3-7.1	302/1,343	0.67 (0.52, 0.86)	68/373	0.60 (0.37, 0.98)	
Q3: 7.1-15.0	277/1,283	0.59 (0.46, 0.76)	49/346	0.54 (0.34, 0.84)	
Q4: ≥15.0	212/1,272	0.47 (0.35, 0.62)	37/309	0.40 (0.22, 0.71)	
p-trend		**<0.001**		**0.005**	
Enterodiol					0.121
Q1: <16.1	333/1,269	Reference	101/489	Reference	
Q2: 16.1-41.9	298/1,298	0.83 (0.66, 1.05)	61/415	0.82 (0.54, 1.26)	
Q3: 41.9-101.4	312/1,282	0.78 (0.61, 1.00)	63/336	1.02 (0.68, 1.54)	
Q4: ≥101.4	257/1,331	0.83 (0.66, 1.04)	57/258	1.23 (0.78, 1.93)	
p-trend		0.331		0.227	
Enterolactone					0.110
Q1: <98.6	343/1,043	Reference	126/475	Reference	
Q2: 98.6-351.2	302/1,286	0.68 (0.54, 0.86)	68/425	0.63 (0.41, 0.96)	
Q3: 351.2-864.4	285/1,395	0.60 (0.48, 0.75)	58/360	0.62 (0.40, 0.95)	
Q4: ≥351.2-864.4	270/1,456	0.63 (0.51, 0.78)	30/238	0.41 (0.23, 0.75)	
p-trend		**0.003**		**0.009**	

OR: odds ratio; CI: confidence interval; O-DMA: O-desmethylangolensin.

* n: the number of individuals with hyperuricemia/normouricemia.

^#^ Adjusted for age, sex, race, body mass index, education level, alcohol intake, hypertension, diabetes, cancer, cardiovascular disease, chronic kidney disease, and liver condition.

Bold p-values indicate statistical significance.

### Nonlinear dose-response associations between urinary phytoestrogens and hyperuricemia

**Fig. 1:**
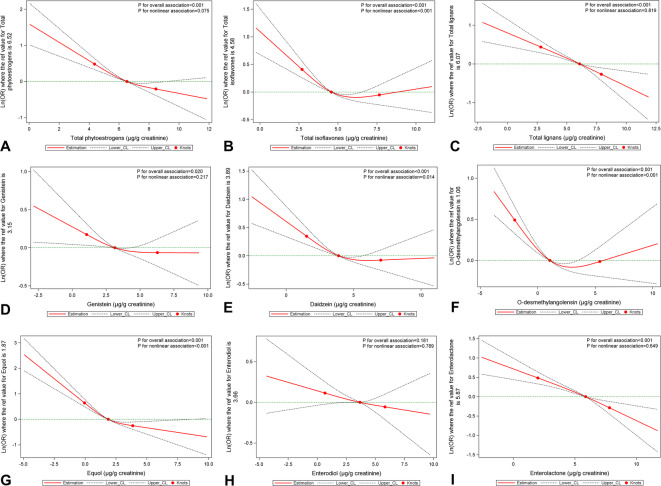
Nonlinear dose-response associations between urinary phytoestrogens and hyperuricemia. Logistic regression models with restricted cubic splines were used to analyze the nonlinear associations. Three spline knots were positioned at the 5th, 50th, and 95th percentiles. Urinary phytoestrogens were log-transformed before analysis. The models were adjusted for age, sex, race, body mass index, education level, cigarette smoking, alcohol intake, hypertension, diabetes, cancer, cardiovascular disease, chronic kidney disease, and liver condition. Y-axis indicates the natural logarithm of the odds ratio (Ln(OR)) for urinary phytoestrogens, compared to their median levels, which were used as the reference point.

Restricted cubic spline curves depict dose–response relationships between urinary phytoestrogens and hyperuricemia risk (Fig. 1). Specifically, L-shaped associations were observed for total isoflavones, daidzein, O-DMA, and equol (all p for nonlinearity < 0.05), with the hyperuricemia risk decreasing sharply at low-to-moderate urinary concentrations and reaching a plateau at higher levels.

## Discussion

Using nationally representative data from U.S. adults, this study demonstrated significant inverse associations of urinary levels of total phytoestrogens, isoflavones, lignans, and several specific compounds with serum uric acid concentrations and the risk of hyperuricemia after adjustment for potential confounders. Among individual phytoestrogens, equol, enterolactone, and O-DMA exhibited the strongest inverse associations. The potential protective effects of total phytoestrogens and isoflavones on hyperuricemia were more pronounced among current smokers. Furthermore, nonlinear analyses showed L-shaped dose–response relationships for total isoflavones, daidzein, O-DMA, and equol, indicating that hyperuricemia risk declined sharply at low to moderate urinary concentrations and stabilized thereafter.

Phytoestrogens, a group of plant-derived compounds with estrogen-like activity, have been reported to provide various metabolic benefits, including improvements in glucose regulation, lipid metabolism, and insulin sensitivity^5,26^. Our findings are generally consistent with previous epidemiological and experimental studies that reported inverse associations between phytoestrogen exposure and serum uric acid or hyperuricemia^12,16–18^. For example, several cross-sectional studies in Asian and U.S. populations found that higher dietary intake of soy isoflavones, including daidzein and genistein, or greater urinary equol concentrations were associated with lower serum uric acid or reduced hyperuricemia risk^16–18^. Similarly, a population-based study reported inverse associations between dietary flavonoid intake, a broader class encompassing certain phytoestrogens, and both serum uric acid concentrations and hyperuricemia risk^17^. Moreover, a meta-analysis of animal intervention studies indicated that supplementation with soy isoflavones, genistein, or daidzein tended to lower serum uric acid concentrations, although the pooled estimate for soy isoflavones did not reach statistical significance^12^. In contrast, a meta-analysis of four human randomized controlled trials (RCTs) with intervention durations ranging from 4 to 24 weeks found no significant effects of soy isoflavones, daidzein, or genistein supplementation on serum uric acid levels^12^.

The inconsistencies in findings between observational studies and RCTs may largely reflect methodological differences. Notably, our findings and those from previous observational studies are consistent with evidence from animal experiments showing that phytoestrogens can lower serum uric acid levels, supporting biological plausibility for this association. Most RCTs were short-term (4–24 weeks) and therefore unlikely to capture the cumulative effects of phytoestrogen exposure on uric acid metabolism. Moreover, RCTs typically use isolated isoflavone supplements, whereas observational studies assess habitual dietary intake or, as in the present study, urinary biomarkers that integrate bioavailability and microbial metabolism. This distinction is critical, as our results showed strong inverse associations for microbial metabolites such as equol, enterolactone, and O-DMA, suggesting that gut microbiota–mediated metabolism plays a pivotal role in determining the biological activity of phytoestrogens. Such interindividual metabolic variability is rarely accounted for in clinical trials, which may partly explain the inconsistent findings^27,28^.

Several biological mechanisms may explain the observed inverse associations between phytoestrogen exposure and hyperuricemia. First, phytoestrogens exert estrogen-like effects by binding to estrogen receptors, which may enhance renal and intestinal excretion of uric acid and consequently lower serum uric acid concentrations^29–31^. Second, certain phytoestrogens, such as genistein, glycitein, and isoformononetin, can directly inhibit xanthine oxidase, the key enzyme catalyzing uric acid formation, thereby reducing uric acid synthesis^13–15,32^. Third, phytoestrogens may modulate the expression of uric acid transporters involved in reabsorption (e.g., URAT1, GLUT9) and excretion (e.g., ABCG2, OAT1), potentially through transcription factors such as c-Jun, leading to enhanced uric acid clearance^32^. Fourth, phytoestrogens possess well-established anti-inflammatory and antioxidant properties^33^, which may help alleviate hyperuricemia by modulating inflammation- and oxidative stress-related pathways^34^. Taken together, these mechanisms provide biological plausibility for the associations observed in this study.

We also observed significant effect modification by smoking status, with stronger inverse associations of urinary total phytoestrogens and isoflavones with hyperuricemia among current smokers. Consistent with our findings, a previous cohort study by our group also observed that the inverse association between dietary isoflavone intake and pancreatic cancer risk was stronger among ever-smokers than among never-smokers^35^, suggesting that smoking may modify the biological effects of phytoestrogens across different disease contexts. This interaction likely reflects the interplay between phytoestrogen exposure and smoking-related oxidative and hormonal changes. Chronic cigarette smoking induces persistent oxidative stress and low-grade systemic inflammation that can jointly impair renal uric acid handling and reduce uric acid excretion^36,37^. Within this pro-oxidative milieu, the antioxidant and anti-inflammatory properties of phytoestrogens may confer greater protection in smokers by counteracting these detrimental effects. In addition, smoking has been reported to exert anti-estrogenic effects and alter sex hormone metabolism^19,38^. Dietary phytoestrogens, as weak estrogen receptor agonists, may partially offset this relative estrogen deficiency and enhance uric acid clearance through estrogen receptor–mediated mechanisms^29^. It is also possible that tobacco smoke alters the intestinal metabolism or bioavailability of phytoestrogens, thereby modifying their biological activity^39^. Together, these findings emphasize that smoking status may modulate the relationship between phytoestrogen exposure and uric acid metabolism, suggesting that smokers, a population at elevated risk for hyperuricemia, may particularly benefit from increasing intake of phytoestrogen-rich foods.

The significant effect modification by smoking status observed for total phytoestrogens and total isoflavones, but not for individual compounds, suggests that overall or combined exposure to multiple phytoestrogens may be more biologically relevant than any single compound. This pattern implies that the collective influence of multiple bioactive compounds, even at modest individual concentrations, could exert a stronger impact on health outcomes^40^. This synergistic effect may arise from the ability of multiple phytoestrogens to act on diverse molecular targets simultaneously, producing broader anti-inflammatory and antioxidant responses than any single compound^41^. Combined phytoestrogens may also modulate estrogen receptor subtypes (ERα and ERβ) more effectively^41^, helping restore signaling pathways disrupted by smoking. Moreover, mixed phytoestrogen intake can overcome the limited bioavailability of individual compounds through complementary metabolic routes, maintaining a higher pool of bioactive metabolites generated by gut microbiota^42^. Collectively, these mechanisms may explain why total phytoestrogens and total isoflavones showed significant interaction with smoking status, whereas individual compounds did not. These observations highlight the importance of evaluating phytoestrogen exposure in an integrated manner in epidemiological studies, particularly among smokers, who may derive greater metabolic benefit from a diet rich in diverse phytoestrogen sources.

A significant interaction by sex was also observed for urinary enterodiol. In the overall population, enterodiol levels were not associated with hyperuricemia; however, stratified analyses revealed an inverse association in males but a positive association in females. These opposite trends may reflect sex-related hormonal differences in uric acid metabolism. Notably, other phytoestrogens did not show such sex-specific patterns, suggesting that enterodiol may have distinct metabolic or receptor-binding properties^43^. As enterodiol is a gut microbiota–derived metabolite of plant lignans, differences in gut microbiota composition and phytoestrogen metabolism between males and females^44^, as well as random variability or residual confounding inherent in population-based studies^45^, may also contribute to these findings. Further epidemiological and mechanistic studies are warranted to confirm this sex-specific association of enterodiol with hyperuricemia and to elucidate the underlying biological mechanisms.

The observed L-shaped dose–response relationships for urinary total isoflavones, daidzein, O-DMA, and equol suggested that hyperuricemia odds decreased sharply at low-to-moderate exposure levels but plateaued at higher concentrations. This nonlinear pattern implied a possible saturation effect, where further increases in exposure yielded minimal additional benefit. Such a pattern may also explain inconsistencies reported in previous studies^12^, as populations with relatively low baseline phytoestrogen exposure are more likely to capture the steep risk reduction phase, whereas those with high habitual intake or using supplemental doses may predominantly reflect the plateau region, resulting in weaker associations. Collectively, these findings highlight that moderate increases in phytoestrogen intake, achievable through typical dietary intake, may confer the greatest population-level benefit in preventing hyperuricemia.

The observed inverse association between urinary phytoestrogen levels and hyperuricemia has important public health implications. Hyperuricemia and its related conditions—gout, hypertension, chronic kidney disease, and metabolic syndrome—represent a growing global health burden^2^. Our findings suggest that higher phytoestrogen exposure, as reflected by urinary biomarker levels and likely influenced by habitual intake of phytoestrogen-rich foods such as soy products, flaxseed, whole grains, and legumes, may play a role in the prevention and management of hyperuricemia at the population level. Given that stronger inverse associations were observed among current smokers, the potential benefits of phytoestrogen-rich diets may be more evident in this subgroup. These findings align with existing evidence supporting the beneficial effects of dietary phytoestrogens on cardiometabolic health^5^. As phytoestrogens are abundant in plant-based foods, these results also suggest that dietary patterns rich in such foods may help reduce the disease burden associated with elevated serum uric acid. Accordingly, public health initiatives promoting diverse phytoestrogen-containing foods may represent a feasible and sustainable dietary strategy for hyperuricemia prevention.

Our study has several notable strengths. The use of nationally representative NHANES data supports that our findings are generalizable to the non-institutionalized U.S. adult population. Moreover, urinary phytoestrogen concentrations were employed as objective biomarkers of exposure, reflecting both integrated dietary intake and host microbial metabolism, while minimizing recall bias inherent in dietary questionnaire–based assessments. To our knowledge, this is among the first comprehensive analyses to examine the associations of urinary total phytoestrogens and specific compounds with hyperuricemia risk, thereby providing novel evidence on the potential contribution of dietary factors to hyperuricemia prevention.

Several limitations should be considered in the present study. First, given the cross-sectional design, the observed associations between urinary phytoestrogen concentrations and hyperuricemia should be interpreted with caution regarding causality. Second, phytoestrogen exposure was assessed using a single spot urine sample, which may not accurately reflect long-term exposure and could lead to non-differential misclassification^46^. Nevertheless, a previous population-based study has demonstrated strong correlations between spot urinary and serum phytoestrogen concentrations (*r* > 0.8)^25^, supporting the utility of this practical measurement approach in large-scale epidemiologic research. Third, despite extensive adjustments for potential confounders, residual confounding due to unmeasured factors (e.g., detailed dietary patterns, gut microbiota composition) may still exist^47^.

## Conclusion

This nationally representative study identified a reduced hyperuricemia risk associated with increased urinary concentrations of total phytoestrogens, isoflavones, lignans, and several specific compounds, with the strongest inverse associations observed for the microbial metabolites equol, enterolactone, and O-DMA. These inverse associations were more evident among current smokers and largely exhibited L-shaped dose-response relationships, suggesting that moderate phytoestrogen exposure may confer the greatest benefit. Future prospective studies with repeated phytoestrogen biomarker measurements are warranted to validate these associations across diverse populations, and experimental studies are needed to elucidate the biological mechanisms underlying the observed effect modification by smoking status.

## Supplementary Information

Below is the link to the electronic supplementary material.

Supplementary Material 1

## Data Availability

All data analyzed in this study are publicly available at https://www.cdc.gov/nchs/nhanes/index.htm.
