# Supplementary material for: Urinary phytoestrogens and hyperuricemia risk in a nationally representative population: an effect modified by smoking status
Source: Sci Rep. 2026 May 6;16:20888. doi: 10.1038/s41598-026-45911-5 (PMC13338021; doi:10.1038/s41598-026-45911-5)
Supplement: Supplementary file 1 — Supplementary Material 1 [file 41598_2026_45911_MOESM1_ESM.pdf]

Supplementary materials

Urinary Phytoestrogens and Hyperuricemia Risk in a Nationally Representative Population: An Effect Modified by Smoking Status

Chunliang Liu, Zhihua Yang, Huizhi Feng, Mengting Liu, Mengrou Zhai, Jiaying Yuan, and Jinliang Niu

Supplementary Figure 1. Flow chart of study participants

Supplementary Table 1. A descriptive overview of the entire study population

Supplementary Table 2. Hyperuricemia risk in relation to urinary phytoestrogens stratified by age

Supplementary Table 3. Hyperuricemia risk in relation to urinary phytoestrogens stratified by sex

Supplementary Table 4. Hyperuricemia risk in relation to urinary phytoestrogens stratified by BMI

Supplementary Table 5. Hyperuricemia risk in relation to urinary phytoestrogens stratified by race/ethnicity

Supplementary Table 6. Hyperuricemia risk in relation to urinary phytoestrogens stratified by history of cancer

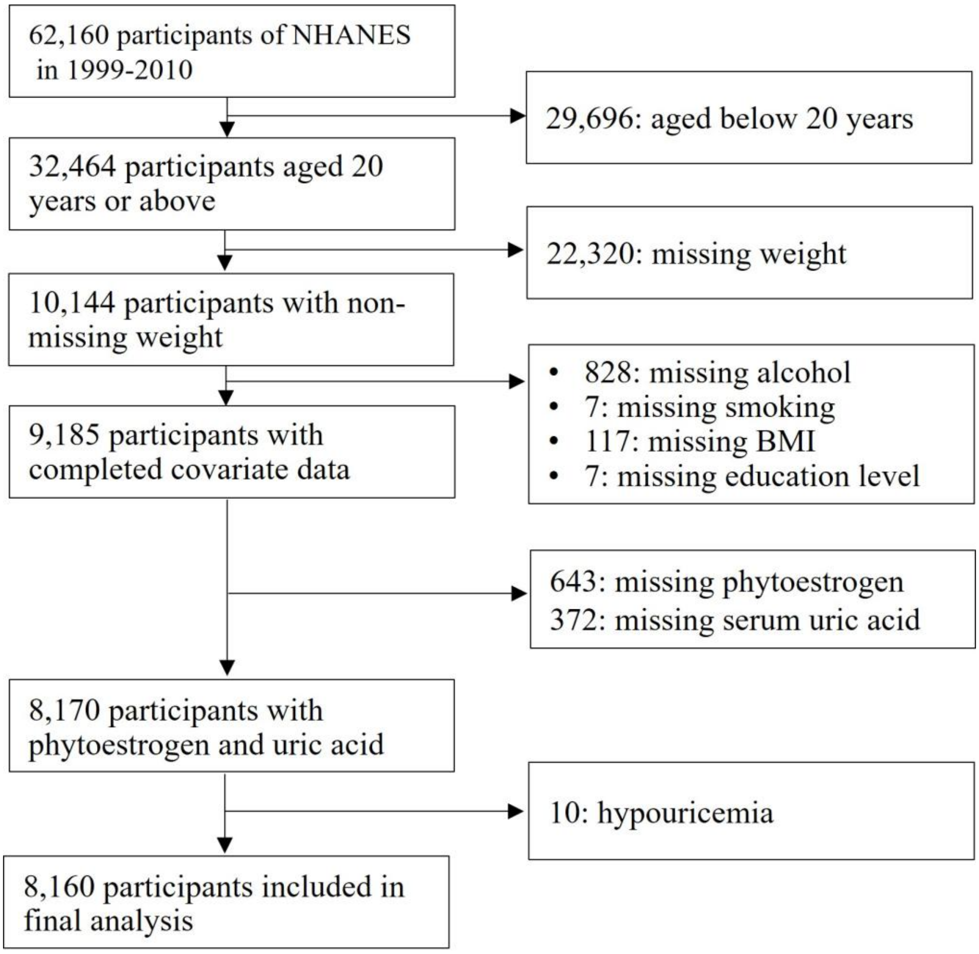

Supplementary Figure 1. Flow chart of study participants

Supplementary Table 1. A descriptive overview of the entire study population

Characteristics	All participants (n = 8,160)
Age, mean (SE)	46.6 (0.30)
Sex [n (weighted %)]	
Male	3,977 (48.74%)
Female	4,183 (51.26%)
Race/Ethnicity [n (weighted %)]	
White, Non-Hispanic	4,111 (72.09%)
Black, Non-Hispanic	1,528 (10.36%)
Mexican American	1,691 (7.89%)
Other Race	830 (9.65%)
BMI (kg/m^2) [n (weighted %)]	
<25	2,418 (32.69%)
25-30	2,838 (32.90%)
≥30	2,904 (34.41%)
Education level [n (weighted %)]	
High school graduate or below	4,292 (43.44%)
Some college or above	3,868 (56.56%)
Cigarette smoking [n (weighted %)]	
Never smoker	4,256 (51.22%)
Former smoker	2,124 (25.06%)
Current smoker	1,780 (23.73%)
Alcohol intake [n (weighted %)]	
Never drinker	1,114 (10.65%)
Former drinker	1,274 (14.41%)
Current drinker	5,772 (74.94%)
Hypertension [n (weighted %)]	2,661 (29.02%)
Diabetes [n (weighted %)]	848 (7.50%)
Cancer [n (weighted %)]	739 (8.73%)
Cardiovascular disease [n (weighted %)]	841 (8.30%)
Chronic kidney disease [n (weighted %)]	184 (1.73%)
Liver condition [n (weighted %)]	249 (3.11%)
Hyperuricemia [n (weighted %)]	1,482 (17.32%)
Serum uric acid (μmol/L) [Median (min–max)]	313.47 (119.00-779.20)
Total phytoestrogens (μg/g creatinine) [Median (min–max)]	700.01 (1.04-142,876.07)
Total isoflavones (μg/g creatinine) [Median (min–max)]	102.63 (0.77-64,712.84)
Total lignans (μg/g creatinine) [Median (min–max)]	433.77 (0.12-142,811.16)
Genistein (μg/g creatinine) [Median (min–max)]	23.59 (0.06-11,524.66)
Daidzein (μg/g creatinine) [Median (min–max)]	49.85 (0.08-58,947.37)
O-DMA (μg/g creatinine) [Median (min–max)]	3.41 (0.02-34,166.67)
Equol (μg/g creatinine) [Median (min–max)]	7.13 (0.01-19,392.16)
Enterodiol (μg/g creatinine) [Median (min–max)]	41.95 (0.01-17,400.00)
Enterolactone (μg/g creatinine) [Median (min–max)]	351.22 (0.08-130,901.29)

Supplementary Table 2. Hyperuricemia risk in relation to urinary phytoestrogens stratified by age

Urinary phytoestrogens (μg/g creatinine)	<50 years		≥50 years		p-interaction
	n*	OR# (95% CI)	n*	OR# (95% CI)	
Total phytoestrogens					0.180
Q1: <317.2	245/1,086	Reference	211/540	Reference	
Q2: 317.2-700.0	133/973	0.77 (0.59, 1.02)	250/722	0.82 (0.61, 1.10)	
Q3: 700.0-1459.5	102/901	0.72 (0.52, 1.00)	252/800	0.69 (0.50, 0.94)	
Q4: ≥1459.5	69/761	0.61 (0.45, 0.84)	220/895	0.62 (0.47, 0.82)	
p-trend		**0.005**		**0.002**	
Total isoflavones					0.077
Q1: <43.5	218/1,065	Reference	240/664	Reference	
Q2: 43.5-102.6	118/896	0.62 (0.46, 0.82)	233/746	0.73 (0.57, 0.95)	
Q3: 102.6-316.3	122/901	0.61 (0.45, 0.83)	222/749	0.72 (0.53, 0.97)	
Q4: ≥316.3	91/859	0.51 (0.38, 0.68)	238/798	0.63 (0.47, 0.85)	
p-trend		**0.001**		**0.028**	
Total lignans					0.281
Q1: <158.3	243/1,043	Reference	224/535	Reference	
Q2: 158.3-433.8	141/1,000	0.71 (0.53, 0.94)	227/677	0.78 (0.58, 1.06)	
Q3: 433.8-980.7	100/931	0.64 (0.46, 0.88)	243/817	0.70 (0.53, 0.92)	
Q4: ≥980.7	65/747	0.66 (0.46, 0.96)	239/928	0.67 (0.51, 0.90)	
p-trend		**0.046**		**0.031**	
Genistein					0.054
Q1: <8.8	192/1,012	Reference	210/643	Reference	
Q2: 8.8-23.6	134/949	0.72 (0.52, 0.98)	233/723	0.75 (0.55, 1.02)	
Q3: 23.6-78.6	125/903	0.68 (0.51, 0.91)	238/772	0.93 (0.71, 1.23)	
Q4: ≥78.6	98/857	0.64 (0.48, 0.85)	252/819	0.81 (0.61, 1.07)	
p-trend		**0.027**		0.443	
Daidzein					0.123
Q1: <17.5	205/1,010	Reference	219/701	Reference	
Q2: 17.5-49.9	136/930	0.76 (0.56, 1.03)	234/692	0.91 (0.69, 1.19)	
Q3: 49.9-170.1	105/900	0.51 (0.36, 0.70)	236/780	0.86 (0.63, 1.17)	
Q4: ≥170.1	103/881	0.60 (0.44, 0.80)	244/784	0.78 (0.59, 1.04)	
p-trend		**0.013**		0.110	
O-DMA					0.054
Q1: <0.7	212/1,065	Reference	250/706	Reference	
Q2: 0.7-3.4	127/920	0.65 (0.47, 0.89)	238/777	0.79 (0.60, 1.03)	
Q3: 3.4-18.9	113/886	0.67 (0.47, 0.95)	213/717	0.66 (0.50, 0.88)	
Q4: ≥18.9	97/850	0.56 (0.42, 0.75)	232/757	0.71 (0.54, 0.95)	
p-trend		**0.007**		0.261	
Equol					0.906
Q1: <3.3	235/1,055	Reference	302/697	Reference	
Q2: 3.3-7.1	129/970	0.62 (0.45, 0.86)	241/746	0.73 (0.55, 0.96)	
Q3: 7.1-15.0	101/886	0.49 (0.35, 0.69)	225/743	0.68 (0.53, 0.88)	
Q4: ≥15.0	84/810	0.49 (0.33, 0.74)	165/771	0.44 (0.34, 0.57)	
p-trend		**0.004**		**<0.001**	
Enterodiol					0.176
Q1: <16.1	195/1,099	Reference	239/659	Reference	
Q2: 16.1-41.9	139/993	0.86 (0.66, 1.12)	220/720	0.80 (0.59, 1.08)	
Q3: 41.9-101.4	127/825	0.95 (0.70, 1.30)	248/793	0.72 (0.55, 0.95)	
Q4: ≥101.4	88/804	0.87 (0.61, 1.23)	226/785	0.89 (0.67, 1.19)	
p-trend		0.587		0.943	
Enterolactone					0.312
Q1: <98.6	237/968	Reference	232/550	Reference	
Q2: 98.6-351.2	144/1,049	0.67 (0.49, 0.91)	226/662	0.73 (0.55, 0.96)	
Q3: 351.2-864.4	105/946	0.63 (0.45, 0.88)	238/809	0.66 (0.49, 0.90)	
Q4: ≥351.2-864.4	63/758	0.62 (0.43, 0.88)	237/936	0.63 (0.47, 0.84)	
p-trend		**0.018**		**0.014**	

OR: odds ratio; CI: confidence interval; O-DMA: O-desmethylangolensin.

* n: the number of individuals with hyperuricemia/normouricemia.

# Adjusted for sex, race, body mass index, education level, alcohol intake, hypertension, diabetes, cancer, cardiovascular disease, chronic kidney disease, and liver condition.

Bold p-values indicate statistical significance.

Supplementary Table 3. Hyperuricemia risk in relation to urinary phytoestrogens stratified by sex

Urinary phytoestrogens (μg/g creatinine)	Male		Female		p-interaction
	n[*]	OR[#] (95% CI)	n[*]	OR[#] (95% CI)	
Total phytoestrogens					0.316
Q1: <317.2	295/869	Reference	161/757	Reference	
Q2: 317.2-700.0	220/838	0.74 (0.57, 0.95)	163/857	0.86 (0.59, 1.24)	
Q3: 700.0-1459.5	189/784	0.63 (0.47, 0.84)	165/917	0.78 (0.54, 1.13)	
Q4: ≥1459.5	145/637	0.62 (0.47, 0.82)	144/1,019	0.60 (0.42, 0.86)	
p-trend		**0.003**		**0.003**	
Total isoflavones					0.145
Q1: <43.5	298/882	Reference	160/847	Reference	
Q2: 43.5-102.6	191/782	0.64 (0.50, 0.83)	160/860	0.69 (0.50, 0.97)	
Q3: 102.6-316.3	198/783	0.67 (0.50, 0.89)	146/867	0.68 (0.51, 0.91)	
Q4: ≥316.3	162/681	0.52 (0.41, 0.67)	167/976	0.63 (0.45, 0.87)	
p-trend		**<0.001**		0.098	
Total lignans					0.609
Q1: <158.3	284/834	Reference	183/744	Reference	
Q2: 158.3-433.8	225/805	0.74 (0.56, 0.97)	143/872	0.72 (0.53, 0.96)	
Q3: 433.8-980.7	181/833	0.58 (0.44, 0.76)	162/915	0.73 (0.53, 1.01)	
Q4: ≥980.7	159/656	0.72 (0.54, 0.96)	145/1,019	0.59 (0.42, 0.82)	
p-trend		0.058		**0.009**	
Genistein					0.063
Q1: <8.8	263/813	Reference	139/842	Reference	
Q2: 8.8-23.6	197/806	0.63 (0.46, 0.84)	170/866	0.91 (0.66, 1.27)	
Q3: 23.6-78.6	210/799	0.79 (0.61, 1.03)	153/876	0.83 (0.61, 1.13)	
Q4: ≥78.6	179/710	0.62 (0.49, 0.79)	171/966	0.87 (0.62, 1.23)	
p-trend		**0.008**		0.656	
Daidzein					0.324
Q1: <17.5	271/855	Reference	153/856	Reference	
Q2: 17.5-49.9	214/769	0.82 (0.62, 1.07)	156/853	0.84 (0.58, 1.22)	
Q3: 49.9-170.1	184/791	0.62 (0.46, 0.84)	157/889	0.77 (0.56, 1.06)	
Q4: ≥170.1	180/713	0.66 (0.51, 0.85)	167/952	0.72 (0.53, 0.97)	
p-trend		**0.012**		0.091	
O-DMA					0.576
Q1: <0.7	295/895	Reference	167/876	Reference	
Q2: 0.7-3.4	202/801	0.68 (0.54, 0.86)	163/896	0.73 (0.49, 1.07)	
Q3: 3.4-18.9	183/764	0.61 (0.46, 0.81)	143/839	0.78 (0.53, 1.14)	
Q4: ≥18.9	169/668	0.61 (0.47, 0.80)	160/939	0.68 (0.48, 0.97)	
p-trend		**0.043**		0.108	
Equol					0.408
Q1: <3.3	347/945	Reference	190/807	Reference	
Q2: 3.3-7.1	198/857	0.60 (0.44, 0.81)	172/859	0.82 (0.58, 1.16)	
Q3: 7.1-15.0	185/702	0.57 (0.41, 0.80)	141/927	0.64 (0.47, 0.87)	
Q4: ≥15.0	119/624	0.45 (0.31, 0.65)	130/957	0.49 (0.35, 0.67)	
p-trend		**<0.001**		**<0.001**	
Enterodiol					**<0.001**
Q1: <16.1	292/928	Reference	142/830	Reference	
Q2: 16.1-41.9	223/881	0.73 (0.58, 0.92)	136/832	1.11 (0.80, 1.54)	
Q3: 41.9-101.4	198/729	0.74 (0.58, 0.95)	177/889	1.07 (0.78, 1.48)	
Q4: ≥101.4	136/590	0.66 (0.50, 0.88)	178/999	1.40 (1.00, 1.98)	
p-trend		**0.025**		**0.036**	
Enterolactone					0.793
Q1: <98.6	269/793	Reference	200/725	Reference	
Q2: 98.6-351.2	238/833	0.75 (0.57, 0.98)	132/878	0.59 (0.42, 0.82)	
Q3: 351.2-864.4	182/814	0.60 (0.46, 0.79)	161/941	0.65 (0.45, 0.93)	
Q4: ≥351.2-864.4	160/688	0.72 (0.55, 0.96)	140/1,006	0.48 (0.34, 0.69)	
p-trend		0.065		**0.001**	

OR: odds ratio; CI: confidence interval; O-DMA: O-desmethylangolensin.

[*] n: the number of individuals with hyperuricemia/normouricemia.

[#] Adjusted for age, race, body mass index, education level, alcohol intake, hypertension, diabetes, cancer, cardiovascular disease, chronic kidney disease, and liver condition.

Bold p-values indicate statistical significance.

Supplementary Table 4. Hyperuricemia risk in relation to urinary phytoestrogens stratified by BMI

Urinary phytoestrogens (μg/g creatinine)	<30 kg/m²		≥30 kg/m²		p-interaction
	n*	OR# (95% CI)	n*	OR# (95% CI)	
Total phytoestrogens					0.478
Q1: <317.2	186/1,008	Reference	270/618	Reference	
Q2: 317.2-700.0	166/1,119	0.63 (0.46, 0.85)	217/576	0.91 (0.69, 1.20)	
Q3: 700.0-1459.5	176/1,192	0.62 (0.43, 0.87)	178/509	0.70 (0.53, 0.92)	
Q4: ≥1459.5	150/1,259	0.53 (0.40, 0.70)	139/397	0.66 (0.50, 0.89)	
p-trend		**0.001**		**0.004**	
Total isoflavones					0.477
Q1: <43.5	218/1,183	Reference	240/546	Reference	
Q2: 43.5-102.6	153/1,129	0.58 (0.43, 0.77)	198/513	0.78 (0.60, 1.01)	
Q3: 102.6-316.3	156/1,101	0.60 (0.43, 0.83)	188/549	0.70 (0.54, 0.91)	
Q4: ≥316.3	151/1,165	0.50 (0.37, 0.68)	178/492	0.64 (0.49, 0.83)	
p-trend		**0.004**		**0.018**	
Total lignans					0.216
Q1: <158.3	191/952	Reference	276/626	Reference	
Q2: 158.3-433.8	166/1,112	0.64 (0.50, 0.82)	202/565	0.80 (0.61, 1.05)	
Q3: 433.8-980.7	158/1,210	0.52 (0.39, 0.70)	185/538	0.72 (0.54, 0.96)	
Q4: ≥980.7	163/1,304	0.55 (0.41, 0.73)	141/371	0.75 (0.57, 0.99)	
p-trend		**0.005**		0.082	
Genistein					0.469
Q1: <8.8	185/1,126	Reference	217/529	Reference	
Q2: 8.8-23.6	165/1,136	0.68 (0.52, 0.89)	202/536	0.73 (0.56, 0.97)	
Q3: 23.6-78.6	162/1,145	0.73 (0.55, 0.97)	201/530	0.84 (0.65, 1.07)	
Q4: ≥78.6	166/1,171	0.63 (0.47, 0.85)	184/505	0.76 (0.57, 1.01)	
p-trend		**0.048**		0.288	
Daidzein					0.586
Q1: <17.5	204/1,190	Reference	220/521	Reference	
Q2: 17.5-49.9	164/1,111	0.74 (0.53, 1.03)	206/511	0.89 (0.66, 1.20)	
Q3: 49.9-170.1	150/1,120	0.59 (0.42, 0.82)	191/560	0.72 (0.55, 0.93)	
Q4: ≥170.1	160/1,157	0.60 (0.45, 0.82)	187/508	0.73 (0.56, 0.95)	
p-trend		**0.026**		0.059	
O-DMA					0.577
Q1: <0.7	226/1,220	Reference	236/551	Reference	
Q2: 0.7-3.4	161/1,140	0.71 (0.53, 0.94)	204/557	0.71 (0.53, 0.94)	
Q3: 3.4-18.9	134/1,092	0.60 (0.44, 0.82)	192/511	0.69 (0.51, 0.94)	
Q4: ≥18.9	157/1,126	0.68 (0.50, 0.91)	172/481	0.59 (0.45, 0.78)	
p-trend		0.262		**0.007**	
Equol					0.072
Q1: <3.3	255/1,187	Reference	282/565	Reference	
Q2: 3.3-7.1	164/1,152	0.67 (0.48, 0.93)	206/564	0.64 (0.49, 0.84)	
Q3: 7.1-15.0	151/1,109	0.57 (0.41, 0.79)	175/520	0.58 (0.43, 0.77)	
Q4: ≥15.0	108/1,130	0.39 (0.28, 0.54)	141/451	0.50 (0.35, 0.71)	
p-trend		**<0.001**		**0.002**	
Enterodiol					0.633
Q1: <16.1	200/1,151	Reference	234/607	Reference	
Q2: 16.1-41.9	158/1,126	0.83 (0.62, 1.11)	201/587	0.84 (0.62, 1.14)	
Q3: 41.9-101.4	153/1,124	0.69 (0.49, 0.96)	222/494	0.99 (0.73, 1.34)	
Q4: ≥101.4	167/1,177	0.88 (0.63, 1.23)	147/412	0.90 (0.65, 1.24)	
p-trend		0.824		0.780	
Enterolactone					0.368
Q1: <98.6	190/933	Reference	279/585	Reference	
Q2: 98.6-351.2	165/1,121	0.62 (0.47, 0.82)	205/590	0.74 (0.56, 0.98)	
Q3: 351.2-864.4	164/1,198	0.55 (0.41, 0.73)	179/557	0.66 (0.49, 0.91)	
Q4: ≥351.2-864.4	159/1,326	0.53 (0.40, 0.69)	141/368	0.68 (0.50, 0.94)	
p-trend		**0.001**		**0.041**	

BMI: body mass index; OR: odds ratio; CI: confidence interval; O-DMA: O-desmethylangolensin.

* n: the number of individuals with hyperuricemia/normouricemia.

# Adjusted for age, sex, race, education level, alcohol intake, hypertension, diabetes, cancer, cardiovascular disease, chronic kidney disease, and liver condition.

Bold p-values indicate statistical significance.

Supplementary Table 5. Hyperuricemia risk in relation to urinary phytoestrogens stratified by race/ethnicity

Urinary phytoestrogens (μg/g creatinine)	Non-Hispanic White		Other races		p-interaction
	n*	OR# (95% CI)	n*	OR# (95% CI)	
Total phytoestrogens					0.820
Q1: <317.2	206/697	Reference	250/929	Reference	
Q2: 317.2-700.0	199/792	0.79 (0.61, 1.03)	184/903	0.69 (0.54, 0.89)	
Q3: 700.0-1459.5	194/878	0.65 (0.49, 0.87)	160/823	0.71 (0.52, 0.96)	
Q4: ≥1459.5	186/959	0.60 (0.45, 0.80)	103/697	0.61 (0.42, 0.88)	
p-trend		**0.002**		**0.025**	
Total isoflavones					0.540
Q1: <43.5	214/676	Reference	244/1,053	Reference	
Q2: 43.5-102.6	182/903	0.61 (0.50, 0.75)	169/739	0.87 (0.63, 1.19)	
Q3: 102.6-316.3	196/874	0.64 (0.49, 0.84)	148/776	0.67 (0.50, 0.90)	
Q4: ≥316.3	193/873	0.57 (0.44, 0.73)	136/784	0.61 (0.45, 0.82)	
p-trend		**0.013**		**0.003**	
Total lignans					0.707
Q1: <158.3	226/699	Reference	241/879	Reference	
Q2: 158.3-433.8	175/785	0.68 (0.53, 0.86)	193/892	0.80 (0.60, 1.06)	
Q3: 433.8-980.7	182/876	0.59 (0.45, 0.77)	161/872	0.69 (0.51, 0.94)	
Q4: ≥980.7	202/966	0.64 (0.48, 0.84)	102/709	0.61 (0.40, 0.94)	
p-trend		**0.027**		**0.027**	
Genistein					0.807
Q1: <8.8	198/748	Reference	204/907	Reference	
Q2: 8.8-23.6	175/829	0.63 (0.48, 0.84)	192/843	1.01 (0.76, 1.34)	
Q3: 23.6-78.6	209/861	0.78 (0.61, 1.00)	154/814	0.79 (0.59, 1.06)	
Q4: ≥78.6	203/888	0.69 (0.54, 0.89)	147/788	0.80 (0.61, 1.05)	
p-trend		0.167		0.131	
Daidzein					0.296
Q1: <17.5	202/759	Reference	222/952	Reference	
Q2: 17.5-49.9	196/827	0.81 (0.61, 1.06)	174/795	0.83 (0.60, 1.13)	
Q3: 49.9-170.1	184/876	0.66 (0.51, 0.86)	157/804	0.66 (0.48, 0.90)	
Q4: ≥170.1	203/864	0.71 (0.55, 0.90)	144/801	0.61 (0.45, 0.82)	
p-trend		0.077		**0.007**	
O-DMA					0.680
Q1: <0.7	203/728	Reference	259/1,043	Reference	
Q2: 0.7-3.4	205/848	0.70 (0.54, 0.93)	160/849	0.72 (0.54, 0.97)	
Q3: 3.4-18.9	188/884	0.67 (0.50, 0.89)	138/719	0.63 (0.45, 0.87)	
Q4: ≥18.9	189/866	0.64 (0.49, 0.85)	140/741	0.63 (0.47, 0.83)	
p-trend		0.074		**0.042**	
Equol					0.213
Q1: <3.3	207/568	Reference	330/1,184	Reference	
Q2: 3.3-7.1	198/785	0.63 (0.47, 0.86)	172/931	0.66 (0.49, 0.89)	
Q3: 7.1-15.0	208/920	0.53 (0.40, 0.71)	118/709	0.73 (0.51, 1.04)	
Q4: ≥15.0	172/1,053	0.41 (0.30, 0.57)	77/528	0.63 (0.42, 0.96)	
p-trend		**<0.001**		0.059	
Enterodiol					0.837
Q1: <16.1	192/707	Reference	242/1,051	Reference	
Q2: 16.1-41.9	191/829	0.77 (0.59, 1.02)	168/884	0.94 (0.71, 1.26)	
Q3: 41.9-101.4	207/896	0.78 (0.60, 1.01)	168/722	0.94 (0.68, 1.29)	
Q4: ≥101.4	195/894	0.88 (0.67, 1.15)	119/695	0.92 (0.65, 1.30)	
p-trend		0.897		0.670	
Enterolactone					0.733
Q1: <98.6	235/699	Reference	234/819	Reference	
Q2: 98.6-351.2	173/787	0.62 (0.49, 0.79)	197/924	0.80 (0.61, 1.03)	
Q3: 351.2-864.4	176/872	0.58 (0.44, 0.76)	167/883	0.66 (0.49, 0.90)	
Q4: ≥351.2-864.4	201/968	0.59 (0.45, 0.78)	99/726	0.58 (0.38, 0.89)	
p-trend		**0.009**		**0.014**	

OR: odds ratio; CI: confidence interval; O-DMA: O-desmethylangolensin.

* n: the number of individuals with hyperuricemia/normouricemia.

# Adjusted for age, sex, body mass index, education level, alcohol intake, hypertension, diabetes, cancer, cardiovascular disease, chronic kidney disease, and liver condition.

Bold p-values indicate statistical significance.

Supplementary Table 6. Hyperuricemia risk in relation to urinary phytoestrogens stratified by history of cancer

Urinary phytoestrogens (μg/g creatinine)	No		Yes		p-interaction
	n*	OR# (95% CI)	n*	OR# (95% CI)	
Total phytoestrogens					0.730
Q1: <317.2	417/1,539	Reference	39/87	Reference	
Q2: 317.2-700.0	330/1,567	0.74 (0.60, 0.90)	53/128	1.32 (0.68, 2.55)	
Q3: 700.0-1459.5	301/1,562	0.65 (0.51, 0.82)	53/139	1.13 (0.64, 2.00)	
Q4: ≥1459.5	245/1,460	0.62 (0.49, 0.77)	44/196	0.68 (0.34, 1.34)	
p-trend		**0.001**		**0.038**	
Total isoflavones					**0.045**
Q1: <43.5	418/1,625	Reference	40/104	Reference	
Q2: 43.5-102.6	306/1,509	0.67 (0.55, 0.81)	45/133	0.65 (0.33, 1.29)	
Q3: 102.6-316.3	297/1,491	0.64 (0.51, 0.82)	47/159	0.75 (0.41, 1.38)	
Q4: ≥316.3	272/1,503	0.55 (0.44, 0.69)	57/154	0.89 (0.45, 1.76)	
p-trend		**<0.001**		0.593	
Total lignans					0.922
Q1: <158.3	422/1,481	Reference	45/97	Reference	
Q2: 158.3-433.8	322/1,570	0.69 (0.57, 0.84)	46/107	1.09 (0.61, 1.93)	
Q3: 433.8-980.7	298/1,595	0.61 (0.49, 0.76)	45/153	0.74 (0.41, 1.33)	
Q4: ≥980.7	251/1,482	0.65 (0.51, 0.82)	53/193	0.66 (0.34, 1.27)	
p-trend		**0.007**		0.151	
Genistein					0.056
Q1: <8.8	371/1,557	Reference	31/98	Reference	
Q2: 8.8-23.6	321/1,539	0.72 (0.58, 0.90)	46/133	0.84 (0.40, 1.77)	
Q3: 23.6-78.6	314/1,530	0.76 (0.62, 0.94)	49/145	1.21 (0.60, 2.43)	
Q4: ≥78.6	287/1,502	0.68 (0.55, 0.86)	63/174	1.24 (0.64, 2.40)	
p-trend		**0.028**		0.339	
Daidzein					0.110
Q1: <17.5	388/1,604	Reference	36/107	Reference	
Q2: 17.5-49.9	328/1,487	0.83 (0.66, 1.05)	42/135	0.64 (0.29, 1.42)	
Q3: 49.9-170.1	288/1,531	0.62 (0.48, 0.78)	53/149	1.24 (0.65, 2.37)	
Q4: ≥170.1	289/1,506	0.66 (0.53, 0.81)	58/159	0.92 (0.45, 1.89)	
p-trend		**0.003**		0.825	
O-DMA					**0.024**
Q1: <0.7	410/1,652	Reference	52/119	Reference	
Q2: 0.7-3.4	325/1,554	0.72 (0.58, 0.89)	40/143	0.55 (0.32, 0.97)	
Q3: 3.4-18.9	287/1,470	0.66 (0.52, 0.85)	39/133	0.59 (0.30, 1.16)	
Q4: ≥18.9	271/1,452	0.60 (0.48, 0.76)	58/155	0.94 (0.51, 1.75)	
p-trend		**0.005**		0.21	
Equol					0.168
Q1: <3.3	477/1,658	Reference	60/94	Reference	
Q2: 3.3-7.1	332/1,573	0.67 (0.53, 0.86)	38/143	0.48 (0.25, 0.91)	
Q3: 7.1-15.0	278/1,466	0.59 (0.46, 0.75)	48/163	0.46 (0.26, 0.82)	
Q4: ≥15.0	206/1,431	0.47 (0.36, 0.62)	43/150	0.34 (0.19, 0.63)	
p-trend		**<0.001**		**0.008**	
Enterodiol					0.633
Q1: <16.1	393/1,657	Reference	41/101	Reference	
Q2: 16.1-41.9	320/1,592	0.82 (0.67, 1.00)	39/121	1.14 (0.54, 2.39)	
Q3: 41.9-101.4	321/1,456	0.82 (0.66, 1.03)	54/162	0.87 (0.49, 1.54)	
Q4: ≥101.4	259/1,423	0.89 (0.70, 1.13)	55/166	1.10 (0.60, 2.00)	
p-trend		0.747		0.822	
Enterolactone					0.707
Q1: <98.6	418/1,414	Reference	51/104	Reference	
Q2: 98.6-351.2	326/1,602	0.65 (0.54, 0.80)	44/109	0.86 (0.48, 1.54)	
Q3: 351.2-864.4	298/1,610	0.58 (0.47, 0.72)	45/145	0.79 (0.42, 1.49)	
Q4: ≥351.2-864.4	251/1,502	0.61 (0.49, 0.77)	49/192	0.54 (0.28, 1.04)	
p-trend		**0.002**		0.065	

OR: odds ratio; CI: confidence interval; O-DMA: O-desmethylangolensin.

* n: the number of individuals with hyperuricemia/normouricemia.

# Adjusted for age, sex, race, body mass index, education level, alcohol intake, hypertension, diabetes, cardiovascular disease, chronic kidney disease, and liver condition.

Bold p-values indicate statistical significance.
